# Ambient air pollution and the risk of violence in primary and secondary school settings: a cross-sectional study

**DOI:** 10.1186/s40621-024-00512-6

**Published:** 2024-06-13

**Authors:** Austin T. Rau, Alyson B. Harding, Andy Ryan, Marizen R. Ramirez, Lynette M. Renner, Jesse D. Berman

**Affiliations:** 1 Division of Environmental Health Sciences, University of Minnesota School of Public Health, Minneapolis, MN 55455 USA; 2 Irvine Program in Public Health, University of California, Irvine, CA 92697 USA; 3 University of Minnesota School of Social Work, St. Paul, MN 55108 USA

**Keywords:** Air pollution, Violence, Weapon, Children, School

## Abstract

**Background:**

Individual and social characteristics are attributed to violent behavior in schools, yet environmental hazards may play an understudied role. Ambient air pollution has been linked to neurological dysfunction that inhibits decision-making and may result in violent behavior in adult populations. However, little is known on how air pollution may be associated with violent behaviors in children.

**Methods:**

A cross-sectional ecologic study was designed to estimate the associations between air pollution (fine particulate matter, carbon monoxide, and nitrogen dioxide) with the occurrence of violent incidents and incidents involving a weapon among a cohort of children in Minnesota schools (2008–2012). Differences by urban and rural status of schools were also explored. Negative binomial regression models were developed to estimate incidence rate ratios (IRR) and incidence rate differences (IRD) to describe associations between air pollution and violent incidents in school settings.

**Results:**

Our results indicate that the highest levels of carbon monoxide, nitrogen dioxide and fine particulate matter concentrations were associated with increased violent disciplinary incidents. Among the total student population, the 4th quartile of carbon monoxide exposure was associated with an IRD of 775.62 (95% CI 543.2, 1008.05) violent incidents per 100,000 students per school year compared to schools in the lowest quartile of exposure. Comparing the 4th to the 1st quartiles of exposure, nitrogen dioxide and fine particulate matter had an IRD of 629.16 (95% CI 384.87, 873.46), and 510.49 (95% CI 274.92, 746.05) violent incidents per 100,000 students per school year respectively. Schools in urban settings shared a larger burden of violent incidents associated with air pollution compared to rural schools.

**Conclusions:**

Modifying environmental pollutants surrounding school environments, particularly for high exposure communities, may be a novel tool for reducing violence and subsequent injuries in schools.

**Supplementary Information:**

The online version contains supplementary material available at 10.1186/s40621-024-00512-6.

## Introduction

Early life exposures to the built and natural environments are critical to consider as they may increase the risk of children’s use of aggressive and violent behaviors which could have ramifications into adulthood. Although drivers of violence are multifaceted, there is a growing amount of research evaluating the contribution of environmental exposures including heavy metals and air pollution on violent activities (Lu 2020; Higney et al. 2022; Lu et al. 2020). Several large, nationwide epidemiological studies in adult populations detected associations between short-term air pollution exposure and an increase in violent criminal behavior, yet the impacts on younger populations have yet to be thoroughly explored (Berman et al. 2019; Burkhardt et al. 2019; Lu et al. 2018).

Although violence can occur in several settings, violence in the school environment is essential to examine because children spend a significant portion of their waking hours at schools. The surrounding school environment may play a key role in children’s overall physical, mental, and emotional development which in turn could affect aggressive behaviors, school violence, and injuries in school settings. Prior research has studied air pollution and academic performance (Mohai et al. 2011; Grineski et al. 2020; Lu et al. 2021; Berman et al. 2018) and absenteeism (Berman et al. 2018; Zhang et al. 2022), but far less research exists on the potential role of air pollution exposure and school violence among children.

Air pollution acts as a toxin and promotes inflammatory responses which may cascade into neuroinflammation, dysregulation and neurodegeneration (Grandjean and Landrigan 2014; Calderón-Garcidueñas et al. 2008; Block and Calderón-Garcidueñas 2009). A stressed neurological system may impair cognitive capacity for active and choice-based decision-making in older adults, and air pollution exposure may also contribute to a rise in neurological diseases including Parkinson’s and dementia (Calderón-Garcidueñas et al. 2008; Block and Calderón-Garcidueñas 2009; Lee et al. 2017). However, the impact on younger populations remains understudied. The limited extant research has shown air pollution exposure may be related to an imbalance in genes involved in oxidative stress, inflammation and other mechanistic hallmarks associated with Parkinson’s and Alzheimer’s disease among urban children (Calderón-Garcidueñas et al. 2013), along with associations between pre-natal and early childhood air pollution exposure with delayed cognitive development (Suglia et al. 2008; Perera et al. 2009; Guxens et al. 2012). Further examination of air pollution associated neurological effects in children should be considered a public health priority as children are particularly vulnerable to adverse health effects from air pollution due to smaller physiques, faster rates of respiration, and developing organ systems (Legot et al. 2012; Gauderman et al. 2007; Garcia et al. 2021; Calderón-Garcidueñas et al. 2014).

The physiological effects of air pollution may vary depending on the exposed hazard. Fine particulate matter (PM_2.5_), comprised of solid and liquid aerosolized particles, can deposit deep into the lungs causing oxidative stress and inflammation in several organ systems including the cardiovascular, respiratory, renal, metabolic and neurological systems (Bont et al. 2022; Li et al. 2019; Burkart et al. 2022; Delgado-Saborit et al. 2021). Other air pollutants, however, target the body via separate pathways. Carbon monoxide (CO) is a colorless, odorless, and tasteless gas that binds exceedingly well with Hemoglobin in the body. Hemoglobin, essential for oxygen delivery, binds with CO 200–300 times more than with oxygen, preventing oxygen from binding to hemoglobin (Patel et al. 2023). This leads to carbon monoxide poisoning in which the body experiences severe hypoxia and may develop symptoms including shortness of breath, headache, an altered mental status, and potentially lead to death (Patel et al. 2023). A study of university students exposed to varying levels of CO revealed even low-level (albeit higher than ambient levels) exposure resulted in impaired cognition and visual processing (Amitai et al. 1998). Due to a sparsity of studies, exact biological mechanisms linking CO and violence are not yet known. Nitrogen dioxide (NO_2_) is a gaseous pollutant, exposure to which can cause irritation of the airways with short-term exposure aggravating respiratory diseases including asthma (Epa 2022). NO_2_ exposure may also affect the neurological system, as researchers have found associations between NO_2_ and dementia (Chang et al. 2014). Considering existing research, it is essential to continue exploring the sources and biological mechanisms through which air pollution affects human health. This line of inquiry is important in furthering our understanding of how air pollution may increase the risk of violent behavior and subsequent injuries in susceptible populations including children.

In this study, we hypothesize ambient air pollution exposure is associated with an increased risk of violent incidents in school settings. To examine this hypothesis, we developed a study to estimate the risk of violent and weapon-related disciplinary incidents among students residing in the state of Minnesota over four academic years. We used air pollution exposure data on three different pollutants (PM_2.5_, NO_2_ and CO) and gathered disciplinary referral data from a comprehensive statewide database to test our hypothesis. In addition, we considered the differences in the associations between schools in urban and rural settings.

## Methods

### Study cohort

We followed all children attending primary and secondary schools (e.g., public, charter, tribal, vocational etc.) in Minnesota across four academic years using data provided through the Minnesota Department of Education (MDE) Minnesota Automated Reporting Student System. Data were collected during the academic years of 2008/09 through 2011/12 from 2681 schools.

### School violence data

We collected the annual number of violent disciplinary incidents among students aggregated at the school-level from the MDE Disciplinary Incident Reporting System (DIRS). We defined violent incidents using several types of incidents captured in the DIRS system that included physical, verbal, and emotional forms of violence (Table S1). In addition to overall school violence, we also studied weapon-related incidents as a subcategory of violent incidents. Violent and weapon-related incidents were expressed as the number of incidents per 100,000 students per school year where a school year was defined as 165 days per Minnesota Statutes, section 120A.41 (n.a. 2022).

### Environmental & community characteristics data

Ambient air pollution data (PM_2.5_, NO_2_, and CO) were collected from the Center for Air, Climate and Energy Solutions (CACES) model at the census tract level (Saha et al. 2021). Air pollution, when studied at an annual time interval, is a slow-moving environmental hazard and concentrations typically do not change drastically from year to year over a short period of time. As such, we used CACES air pollution data for 2010 in our primary analysis because it aligned with the midpoint of our study period.

The 2010 Social Vulnerability Index (SVI) was obtained at the census tract level from the Centers for Disease Control and Prevention as a measure of overall community vulnerability (Centers for Disease Control and Prevention/Agency for Toxic Substances and Disease Registry/Geospatial Research, Analysis, and Services Program 2010). The SVI is a composite index that describes community vulnerability to external stressors. An overall SVI score and four sub-theme SVI scores are derived from census metrics of demographic, economic, housing and transportation variables (CDC 2010). County-level total crime rates for 2015 were obtained from the Minnesota Bureau of Criminal Apprehension as a measure of overall community violence (Bureau of Criminal Apprehension, Criminal Justice Information Systems Section. Minnesota crime information [Uniform crime report] 2015). The 2010 crime data were not used because data reported prior to 2015 were not readily available. Additionally, statewide crime data were not available at geographies smaller than the county-level. For the environmental variables, geocoded point locations of schools in Minnesota were spatially overlaid and assigned the value of the census tract they fell within (or county for crime data).

The 2010 Rural–Urban Area Commuting Codes (RUCA) at the census tract level from the U.S. Department of Agriculture Economic Research Service (U.S. 2010) were used to assign measures of urbanicity to the schools in our study. From these RUCA codes, we created a binary urban–rural stratification using guidance from the Washington State Department of Health Guidelines for Using Rural–Urban Classifications Systems for Community Health Assessment scheme #1 where urban schools were RUCA codes 1.0, 1.1, 2.0, 2.1 and 3.0 and rural schools were all other RUCA codes (Washington State Department of Health 2016).

### Study design

We designed a cross-sectional ecologic study to measure the associations between ambient air pollution (PM_2.5_, NO_2_, and CO) and school-level violence and weapon specific incidents using four academic years (2008/09–2011/12) of data aggregated at the school-level.

### Statistical analysis

Negative binomial regression models with a random intercept for schools and an offset for the total number of school years (person-time) were developed to estimate incidence rate ratios (IRR) describing the association between ambient air pollution and rates of violence in single pollutant models. Incidence rate differences (IRD) were calculated via statistical contrasts using the R package *emmeans* (Lenth 2022). For each pollutant, air pollution exposure was expressed as quartiles with the first quartile set as the referent. Models were adjusted for census tract level overall SVI (quartiles), year (linear term) and county-level total crime rates (linear term). Effect measure modification was evaluated for the urbanicity of schools using stratified data analyses for urban and rural schools with recalculated quartiles of pollutant exposure specific to these subpopulations. As a secondary analysis, we generated multi-pollutant models in which the effect estimates for each pollutant were adjusted for the other two pollutants.

All statistical analyses were completed in R statistical software (V 4.1) (R Core Team. R 2021).

### Sensitivity analysis

We completed sensitivity analyses to evaluate our model robustness against alternative exposure definitions and confounding adjustments. First, we calculated the mean annual ambient air pollution concentrations during 2008–2012 for each pollutant and created quartiles of exposure to use as the primary exposure variable compared with our original analysis that used 2010 CACES data. Second, we compared our model estimates using the socioeconomic status SVI measure instead of the overall SVI to adjust more specifically for socioeconomic status rather than overall community vulnerability. Third, we completed an analysis stratifying schools based on the minority status SVI measure to assess whether schools in diverse communities were at greater risk of air pollution associated violence. Finally, we compared model outcomes for three other subcategories of violence to those for weapons-related violence: (1) physical violence (fighting or assault or robbery using force), (2) verbal/threat (verbal abuse or threat/intimidation), and (3) vandalism/property related. All sensitivity analyses were completed for total violent incidents in the total population.

## Results

### Descriptive statistics

Over the study period, there were 2,681 primary and secondary schools (1671 urban and 1010 rural schools) with a total of 3,492,049 enrolled students contributing approximately 4.8 million school years of person-time (Table 1). A total of 831,002 students had a violent or weapon-related incident reported in DIRS. More students attended urban schools than rural schools and incidence rates of violent and weapon-related incidents were higher in urban schools than rural schools (Table 1).

**Table 1:** Summary of violent and weapon-related disciplinary incidents (2008–09 to 2011–12) in 2681 Minnesota schools

Strata	Student cohort^A^	Student school years^B^	Violent incidents^C^	Weapon-related incidents^C^
Total	831,002	4,875,794	89,860 (1,842.98)	4525 (92.81)
Urban	609,570	3,579,306	71,554 (1999.10)	3410 (95.27)
Rural	241,417	1,296,488	18,306 (1,411.97)	1115 (86.00)

^A)^Total number of unique students who had a disciplinary referral for a violent incident. Urban and rural student categories will not add up to the total student cohort because students may have switched school types during the study period

^B)^A school year in Minnesota equals 165 instructional days and we included 4 academic years in this study

^C)^Total number of incidents (incidence rate per 100,000 students per school year)

**Fig. 1:**
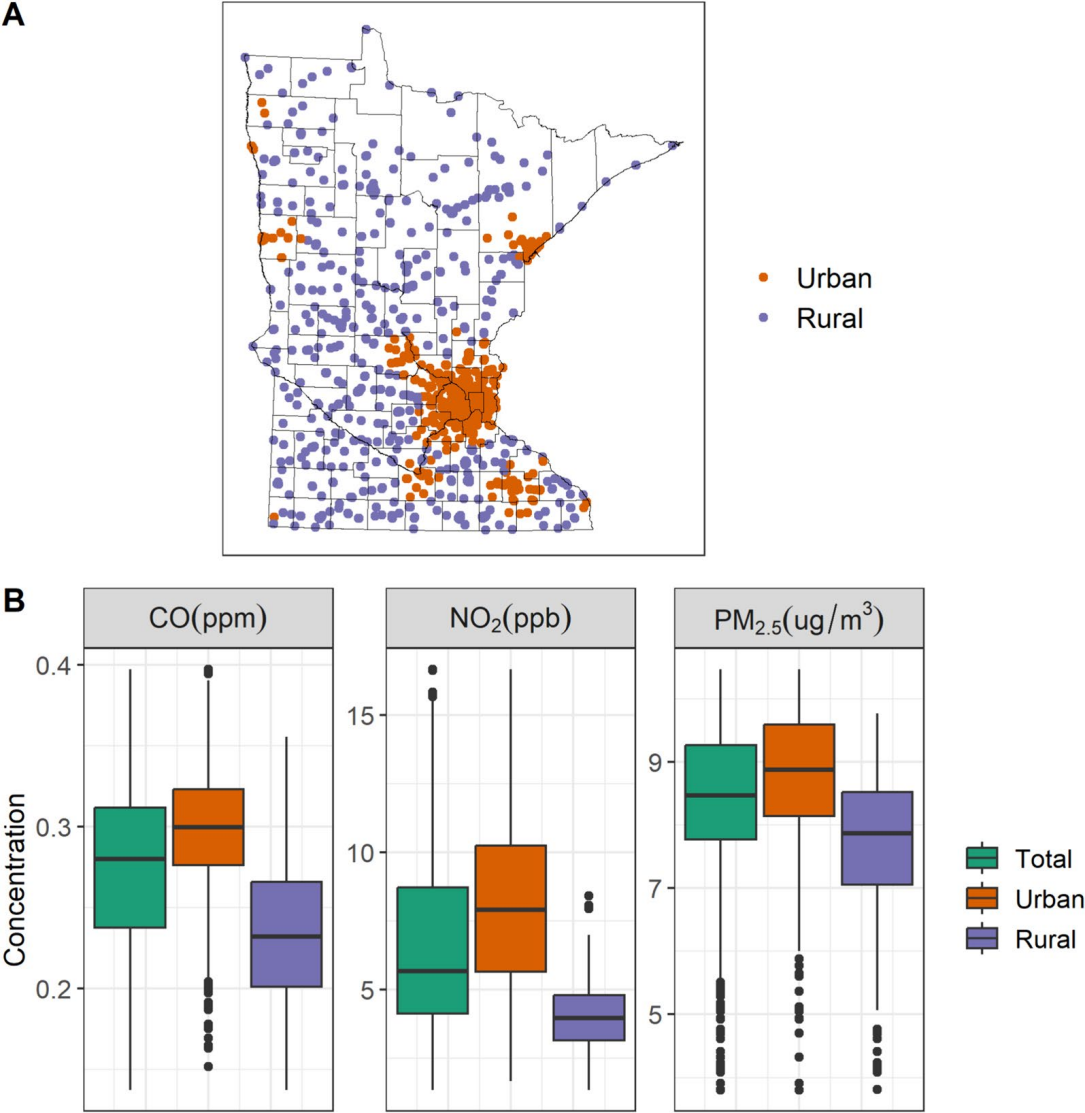
School locations stratified by urban and rural status (**A**). Air pollution concentrations (2010) stratified by urban and rural status (**B**)

Quartiles of air pollutants were higher in urban schools compared to rural schools (Figure S1) with large differences between the 4th quartiles of exposure for all pollutants (Fig. 1, Table S2).

**Fig. 2:**
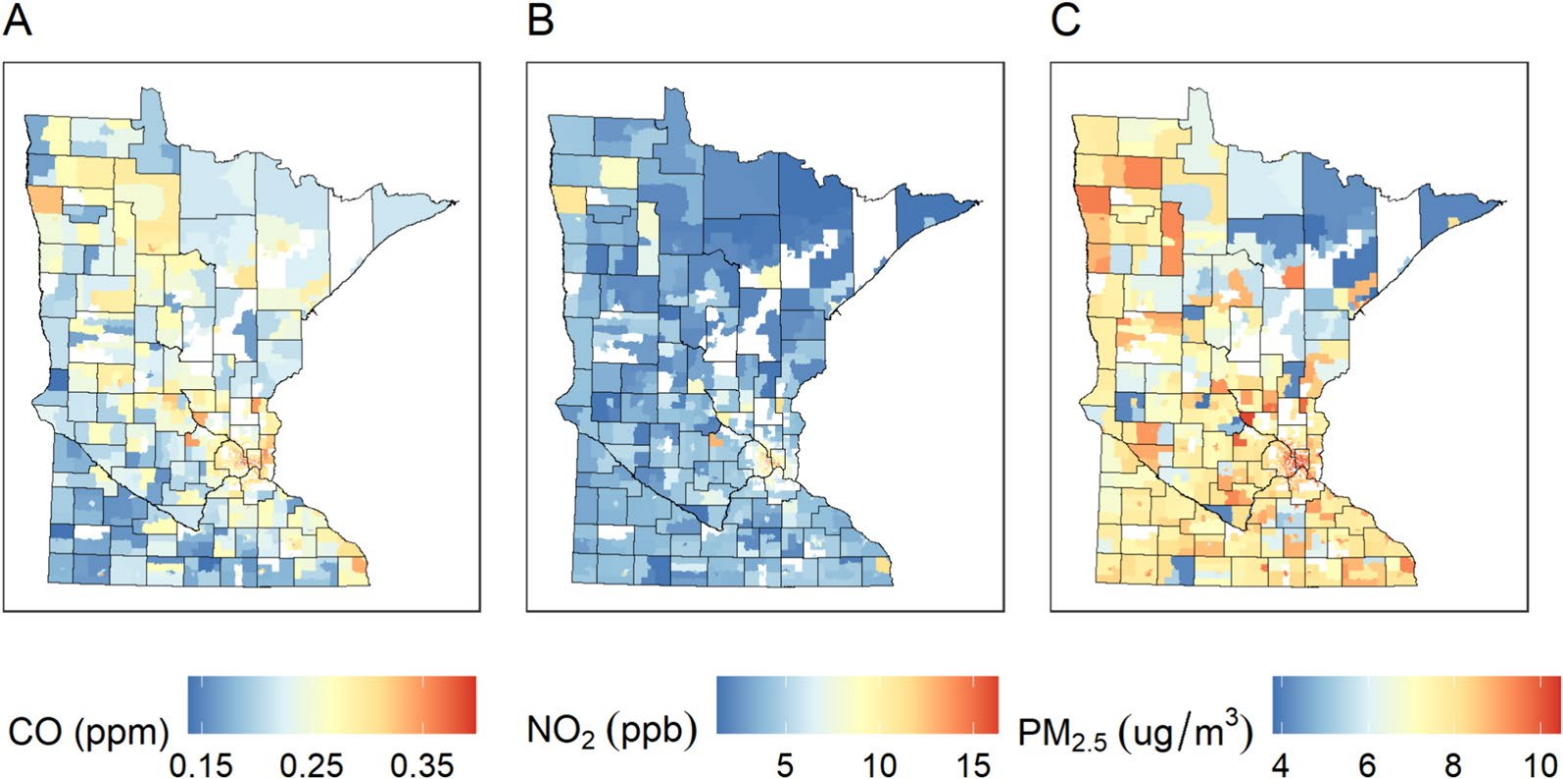
2010 Census tract level air pollution concentrations CO (**A**), NO_2_ (**B**), PM_2.5_ (**C**). Unshaded census tracts did not contain a school in our study cohort

The spatial distribution of ambient air pollution in Minnesota indicated higher levels of CO and NO_2_ primarily in the Twin Cities 7-county metropolitan area (Fig. 2). Elevated PM_2.5_ was observed in several locations throughout the state with low concentrations in the northern arrowhead (Fig. 2).

**Fig. 3:**
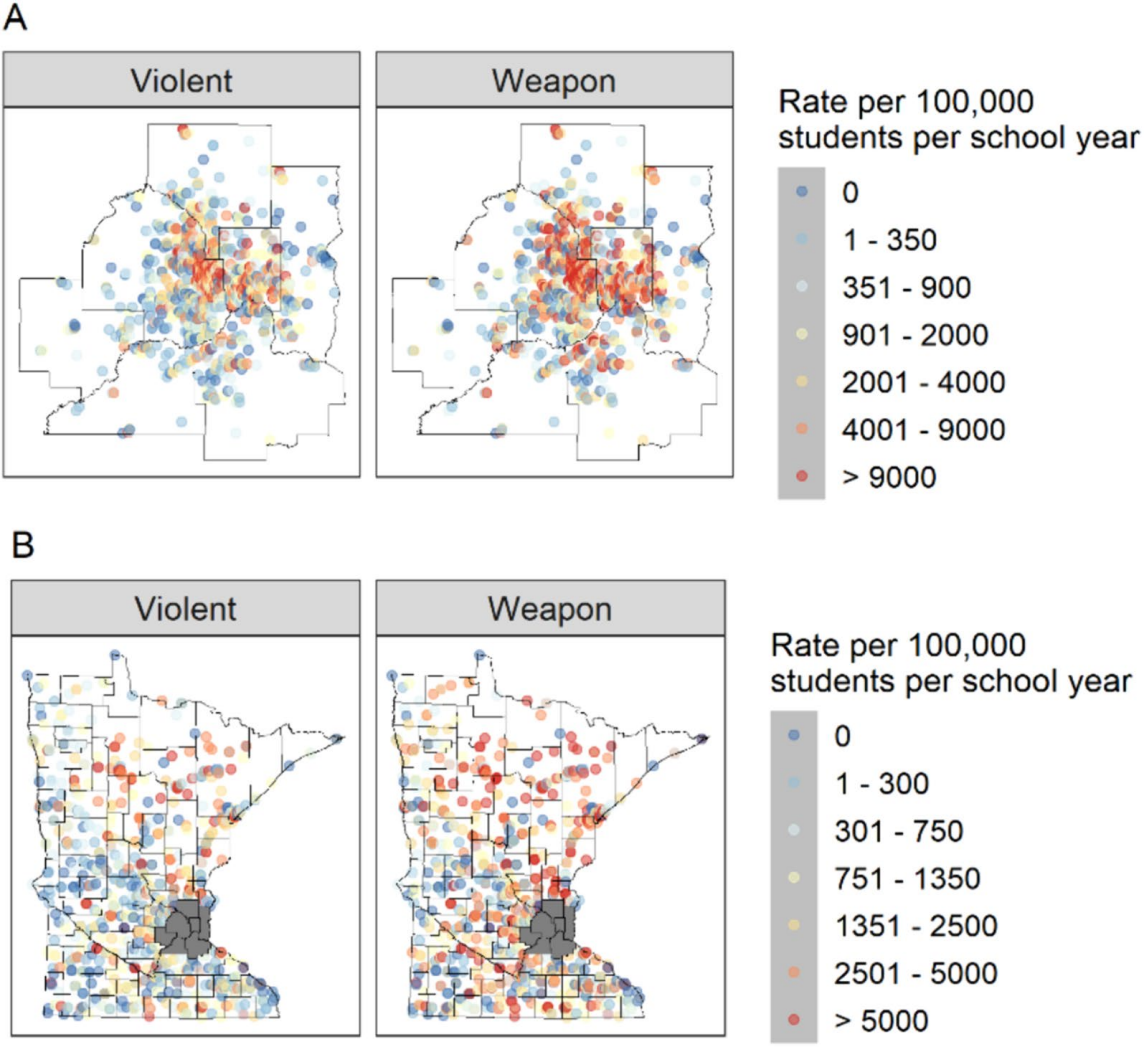
Average incidence rates per 100,000 students per school year of violent and weapon-related incidents during the 2008–09 to 2011–12 academic years. Panel **A** shows schools in the 7-county metropolitan area and panel **B** shows all schools outside of the Twin Cities 7-county metropolitan area (shaded gray)

There was a heterogeneous spatial distribution of violent disciplinary incidents across urban and rural schools (Fig. 3). In urban schools, high levels of violent incidents were concentrated in the Twin Cities 7-county metropolitan area whereas for schools outside of the Twin Cities 7-county metropolitan area, the western part of the state had relatively lower rates (Fig. 3).

### Air pollution and violence

Crude estimates indicated the 4th quartiles of air pollution exposure in the total population of students had the strongest associations with violent incidents, while the 3rd and 2nd quartiles tended to have null or protective associations (Table S3). Effect estimates from adjusted single pollutant models were attenuated compared to the crude model results (Table S4). However, the 4th quartiles of pollution exposure for CO, NO_2_ and PM_2.5_ retained positive associations with violent incidents in the total population (Table S4).

**Fig. 4:**
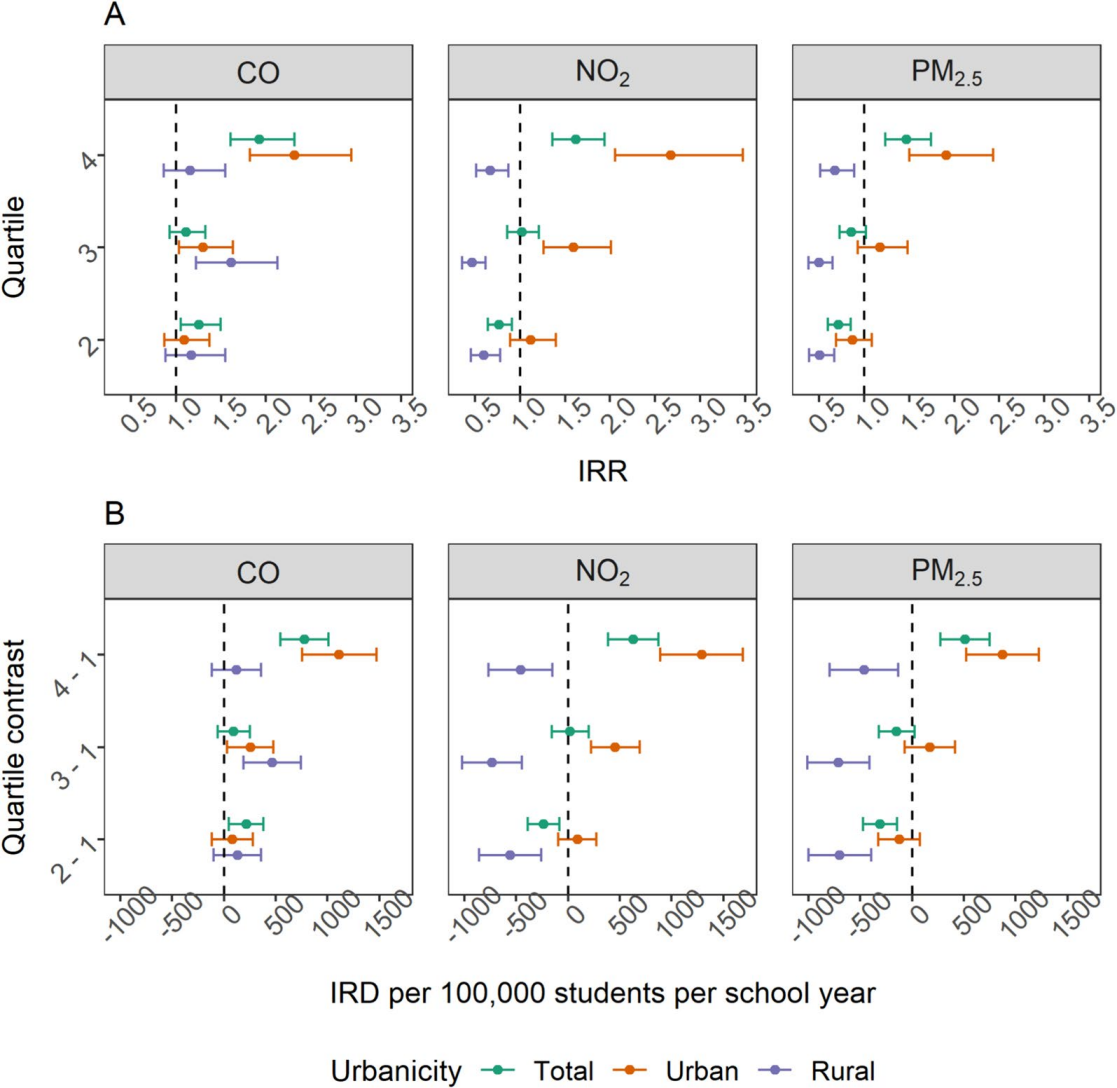
Adjusted incidence rate ratios (IRR) (**A**) and incidence rate differences (IRD) (**B**) describing associations between air pollution concentrations with violent incidents with comparisons made between schools in the lowest pollutant quartile (e.g., 1st quartile) and other quartiles of pollution. Pollutant quartiles were drawn within the total, urban, and rural schools respectively

Results from adjusted models indicated the 4th quartiles of exposure for all pollutants under study were associated with an increased risk of violent incidents in school settings among the total student population (Fig. 4). The 4th quartile IRD estimates had a range from 510.49 to 775.62 violent incidents per 100,000 students per school year (Table S4). The highest IRD estimate was associated with 4th quartile CO exposure (Table S4).

Among urban schools, the 4th quartiles of all pollutants were associated with an increased occurrence of violent incidents (Fig. 4). The 4th quartile IRD estimates ranged from 874.91 to 1,288.88 violent incidents per 100,000 students per school year (Table S4). The greatest frequency of violent incidents was attributed to the 4th quartile of NO_2_ exposure (Table S4). Rural schools had null and protective associations with quartiles of PM_2.5_ and NO_2_ exposure (Table S4). CO was the only pollutant that conferred an increased risk of violence among rural schools, and it was the 3rd quartile of exposure that had the largest IRD of 464.44 (95% CI 187.21, 741.66).

Results from multi-pollutant models were moderately attenuated compared to their single pollutant counterparts (Table S5, Figure S2). In both the total population and urban schools specifically, only NO_2_ and CO exposure maintained significant positive associations with violent incidents for most exposure quartiles (Table S5). PM_2.5_ had null associations (Table S5). The rate of violent incidents in rural schools was not positively associated with any pollutants in the multi-pollutant models (Table S5).

### Air pollution and violence involving a weapon

**Fig. 5:**
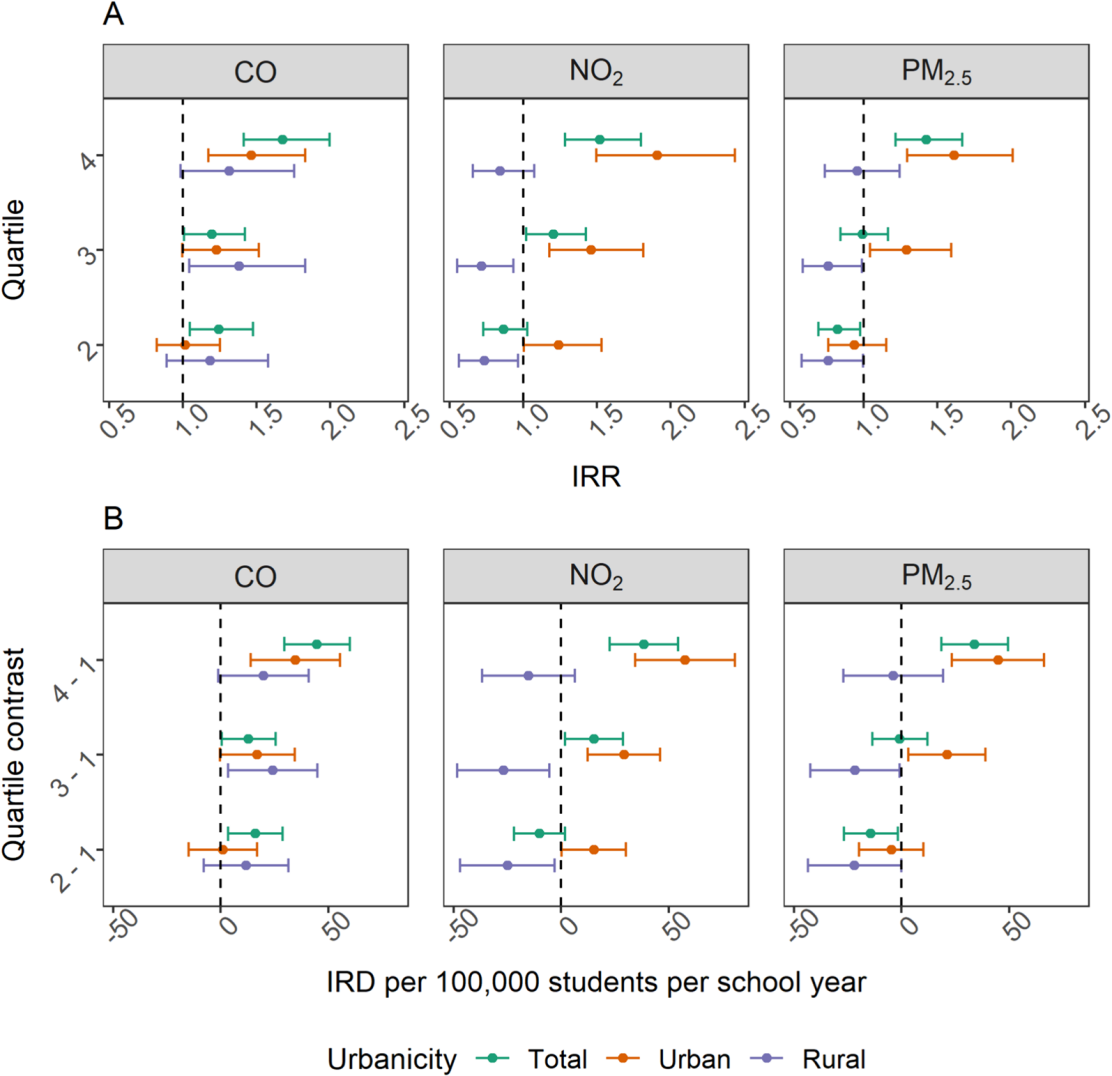
Adjusted incidence rate ratios (IRR) (**A**) and incidence rate differences (IRD) (**B**) describing associations between air pollution concentrations with weapon-related incidents with comparisons made between schools in the lowest pollutant quartile (e.g., 1st quartile) and other quartiles of pollution. Pollutant quartiles were drawn within the total, urban, and rural schools respectively

In adjusted models, the 4th quartiles of exposure for all pollutants were associated with an increased risk of violent incidents involving a weapon among the total population of students (Fig. 5). The IRD estimates for the 4th quartiles of air pollution exposure ranged from 33.96 to 44.84 weapon-related incidents per 100,000 students per school year. The 4th quartile of CO had the largest increase in weapon-related incidents (Table S4). All quartiles of CO exposure (Q2–Q4) increased the risk of weapon-related incidents (Table S4).

Within urban schools, the 4th quartiles of pollution exposure were associated with an increase in the rate of weapon-related incidents ranging from 44.90 to 57.64 weapon-related incidents per 100,000 students per school year (Fig. 5). The 4th quartile of NO_2_ conferred the largest increase in the frequency of weapon-related incidents (Table S4). In rural settings, NO_2_ and PM_2.5_ had null associations with weapon-related incidents (Table S4). CO was the only pollutant associated with an increased risk of weapon-related incidents in rural schools, and the largest risk was seen in the 3rd quartile of CO exposure, IRD: 24.25 (95% CI 3.38, 45.11).

Similar to the results for all violent incidents, the single pollutant model estimates for weapon-related incidents were attenuated under multi-pollutant models (Table S5, Figure S3). In the total population, the 4th quartile of NO_2_ and CO exposure maintained positive associations with violent incidents involving a weapon, while PM_2.5_ had null associations (Table S5). Among urban schools, all quartiles of NO_2_ exposure maintained positive associations. Violent incidents involving a weapon were not positively associated with any pollutants in the multi-pollutant models among rural schools (Table S5).

### Sensitivity analysis

Our findings were unchanged when comparing model estimates adjusted using the overall SVI versus the socioeconomic specific component of the SVI in the total student population (Table S6). In models adjusted using the socioeconomic SVI, the risk of violent incidents associated with air pollution increased slightly compared to our model adjusted for overall SVI (Table S6). When comparing models using air pollution exposure measured during 2010 (our primary analysis) against using average air pollution concentrations from 2008 to 2012, our estimates were changed such that CO was no longer the pollutant with the largest association with violent incidents among the total population (Table S7). Rather, the 4th quartile of NO_2_ exposure conferred the greatest risk of violent incidents in the overall population. However, the magnitude of risk for both CO and NO_2_ were similar (Table S7). Estimates of CO may need to be interpreted cautiously. Results stratified by the minority status SVI component suggested schools in more diverse communities had greater risks of air-pollution associated violence compared to less diverse communities (Figure S4). Future investigations with more robust measures of structural racism are needed to examine these disparities in more depth. Finally, results for three other subcategories of violence (physical, verbal/threat, and vandalism/property related) were similar to those for weapons-related violence; the 4th quartile of pollutant exposure was associated with an increased risk for each type of violence (Figure S5).

## Discussion

Our study was among the few designed to measure associations between school-level air pollution and violent incidents in primary and secondary schools. In single pollutant models, we found that the highest concentrations of CO, NO_2_ and PM_2.5_ were all associated with aggressive and violent behaviors in youth. For all pollutants studied, exposure exceeding the 4th quartile (CO: 0.28 ppm, NO_2_: 5.64 ppb, and PM_2.5_: 8.46 μg/m^3^) was associated with a greater risk of violent incidents and incidents involving a weapon in a population of K-12 Minnesota students. These associations were detected despite the 4th quartile thresholds for all pollutants being beneath the Environmental Protection Agency’s (EPA) annual regulatory limits (The 4th quartile of PM_2.5_ in our study was below the prior annual standard of 12 µg/m^3^. The regulatory standard for PM_2.5_ was lowered to 9 µg/m^3^ by EPA on February 7, 2024) (Epa and O. NAAQS 2024). Lower exposures (quartiles 2 and 3) had mostly null or protective associations, which suggests that there may be a threshold at which pollution engenders greater risk of school violence. This has potentially important implications for future decision-making regarding air pollution exposures surrounding schools.

Our results found CO, an understudied pollutant that can have severe neurological effects, had the largest associations with the occurrence of violent incidents in schools. Research on the connection between CO and violence is lacking, but our findings were congruent with other research that found positive associations between CO and mental health emergency department visits (related to homicide/inflicted injuries) in adults (Thilakaratne et al. 2020), and behavioral changes in the form of internalizing behavior (e.g. anxiety, depression) among children (Qi et al. 2023). Although, our results were not congruent with Wesselbaum (2022) who found a U shaped relationship between CO and violent crime (Wesselbaum 2022). Rather, our findings suggest a possible threshold of CO exposure at which violent behaviors may occur. Although the mechanisms relating CO to externalizing behaviors (e.g. hyperactivity, aggression) have yet to be fully understood, CO exposure is known to have deleterious health effects including dyspnea, headache, and an altered mental status (Patel et al. 2023), each of which may affect the mood of children and possibly lead to aggressive behavior.

We additionally found an increased risk of school violence associated with high levels of NO_2_, a pollutant closely related to traffic and industrial combustion. Previous studies demonstrated associations between NO_2_ and internalizing and externalizing behaviors along with general psychopathology in children (Loftus et al. 2020; Reuben et al. 2021). NO_2_ exposure has also been linked to an increased risk of self-harm among children (Mok et al. 2021), and with mental health emergency visits among children and adolescents (Szyszkowicz et al. 2020). Given the findings of previous studies, it is plausible that high levels of NO_2_ exposure may affect the mental status of students in such a way that violent behavior and possible injuries could occur. Our findings highlight the harmful role that traffic related air pollution may play in child development and necessitate increased attention to the location of schools with respect to large roadway systems.

Of the three pollutants analyzed in our study, PM_2.5_ is among the most well-represented in the literature focused on air pollution and behavior/violence (Berman et al. 2019; Burkhardt et al. 2019; Reuben et al. 2021; Szyszkowicz et al. 2020; Haynes et al. 2011). Elevated PM_2.5_ exposure has been linked with increased risk of emergency department visits among adolescents with a mental health disorder diagnosis (Szyszkowicz et al. 2020), and an increase in the risk of self-harm in youth (Mok et al. 2021). Similar to CO and NO_2_, the inflammatory neurological responses from toxic particulate matter exposure may propagate into aggressive behavior among students. Compared to previous research, the associations we found between the 4th quartile of PM_2.5_ and violence were relatively larger than associations detected in other studies. For example, Haynes et al. (2011) found a 12% increase in juvenile adjudication rates for each natural logarithm increase in PM_2.5_ (Haynes et al. 2011), while Berman et al. (2019) found a 1.17% increase in risk for each 10μ/m^3^ increase in PM_2.5_ (Berman et al. 2019). Among the total population of students in our study, we found a 47% increase in the risk of school violence for schools in the most polluted quartile of exposure compared to the least polluted schools. The reasons behind our relatively large measure of association may be driven by differences in study design, exposure windows, or the population under study.

The results of our urban stratified analyses were consistent with our expectations for urban schools but had unanticipated results for rural schools. It is unsurprising that urban schools were most affected by NO_2_ because vehicle emissions are a primary source for this pollutant. Commuting patterns in the Twin Cities 7-county metropolitan area may be contributing to high levels of NO_2_ in urban areas (Pratt et al. 2015) and subsequent increases of violence in urban schools. Previous research found surrounding suburban communities with larger shares of vehicle ownership imported air pollution into the urban core of the Twin Cities area whose residents had lower rates of car ownership and contributed less to traffic related pollution (Pratt et al. 2015). Rural areas have been historically understudied in public health research of air pollution due to low population sizes and sparsity of air monitors in rural settings. Our results of air pollution associated violence in rural areas were unexpected with higher levels of PM_2.5_ and NO_2_ having seemingly protective associations with violence. This is counterintuitive to our understanding of pollution and health and could be reflective of potential shortcoming of air pollution models in rural areas. It is imperative to invest more time and resources into studying violence and health disparities in rural communities, so they do not lag behind urban areas in achieving meaningful progress in reducing environmental pollution and school violence.

Our multi-pollutant models had reduced magnitudes of associations compared to our single pollutant models, to the extent where nearly all associations were null or protective. However, it is not uncommon for multi-pollutant models to produce estimates that are lower compared to single pollutant models (Berman et al. 2019; Thilakaratne et al. 2020; Gold et al. 1999; Winquist et al. 2014). Interestingly in our study, multi-pollutant models for NO_2_ and CO had persistent, robust associations with violence among students in urban schools. This finding is contrary to Thilakaratne et al. (2020) who found NO_2_ did not have associations with emergency department visits (related to homicide/inflicted injuries) in multi-pollutant models yet CO did (Thilakaratne et al. 2020).

Although the multi-pollutant results offer a glimpse into the associations between air pollution and violence in a more real-world context of pollutant mixtures in the environment, we chose to emphasize the results of our single pollutant models as the most relevant to public health policy and regulations. Multi-pollutant models suffer in treating co-pollutants as confounders when they may have compounding synergistic effects which are methodologically difficult to capture. From a regulatory standpoint, multi-pollutant models can muddy the interpretations of pollution and health because of the complex interactions of pollutants with each other. Single pollutant models allow for the generation of isolated and targeted regulatory actions and public health guidance for a specific pollutant. Additionally, pollutants have different physiological impacts on the human body with potentially disparate health effects which necessitates the use of single pollutant models.

Interventions to mitigate air pollution exposure around schools may include policies regulating where sources of pollution can be located in proximity to new and existing schools. These types of regulations may have important implications for racial and economic equity as was seen in our minority status SVI stratification analysis. Historically marginalized communities are disproportionately impacted by environmental pollution and suffer the brunt of severe health outcomes from toxic exposures. Living in high poverty neighborhoods has been associated with increased air pollution exposure in early childhood and reduced cognitive ability by 0.10 standard deviations by age four (Wodtke et al. 2013). Furthermore, schools with higher proportions of students of color and students on free and reduced lunch programs have disproportionately higher exposure to air pollution and proximity to sources of lead emissions (Oliva and Som 2022; Cheeseman et al. 2022). Additional mitigation efforts such as retrofitting equipment at existing industrial complexes and building physical infrastructure along heavily trafficked roads are other measures which could be implemented to reduce air pollution exposure in schools. More importantly, larger systemic changes should be considered to address the upstream causes of air pollution exposure. These structural changes may include lowering air quality standards, transitioning to clean energy sources, improving public transportation and investing in improving the infrastructure within the most disadvantaged schools.

The primary limitation of our study was the ecological data used to evaluate associations between air pollution and school violence. We were unable to adjust for individual level covariates which may have confounded the relationships presented, and our results can only inform school-level risks. We were also limited in the temporal resolution of the violence data which were annual counts. This impeded the detection of seasonal or short-term trends in violence associated with air pollution which has well-known seasonal fluctuations in concentrations. Rather, our study provided information on the association of long-term air pollution exposure and violence among children.

Additionally, there may have been some exposure misclassification because the air pollution data was acquired at the census tract level which can be variable in size with urban areas having small census tracts and rural areas having large census tracts in general. Despite these limitations, our study analyzed data from a unique statewide cohort of children and identified statistically precise measures of association between ambient air pollution and school violence.

## Conclusion

Environmental pollution is often overlooked in research focused on aggressive behavior and violence. Yet, as our results suggested, high levels of air pollution may be associated with a relatively large increase in the frequency of violent incidents in primary and secondary schools, which could lead to a greater occurrence of injuries in school settings. Reductions in the highest exposures to air pollution are critical not only to improve children’s health and reduce school violence, but also to prevent children from future involvement in violent activities. Addressing upstream effects and early life exposure to environmental hazards is essential to reducing the public health burden of violence and injuries in society. Although challenging, successful efforts to enact changes to improve environmental conditions at schools can be achieved with engagement from researchers, policymakers, advocacy groups, and community members.

### Supplementary Information

Supplementary Material 1.

## Data Availability

This study used data housed in the Minnesota Linking Information for Kids (Minn-LInK) project at the Center for Advanced Studies in Child Welfare (CASCW) at the University of Minnesota. The datasets generated and analyzed during the current study are not publicly available, but arrangements to access data may be made by contacting the CASCW Director of Research and Evaluation at cascw@umn.edu.
