# Supplementary material for: Ambient air pollution and the risk of violence in primary and secondary school settings: a cross-sectional study
Source: Inj Epidemiol. 2024 Jun 13;11:24. doi: 10.1186/s40621-024-00512-6 (PMC11170797; doi:10.1186/s40621-024-00512-6)
Supplement: Supplementary file 1 — Supplementary Material 1. [file 40621_2024_512_MOESM1_ESM.docx]

**Supplemental Materials to**

**Ambient air pollution and the risk of violence in primary and secondary school settings: a cross-sectional study**

Austin T Rau^1^, Alyson B Harding^1^, Andy Ryan^1^, Marizen R Ramirez^1,2^, Lynette M Renner^3^, Jesse D Berman^1^

^1^University of Minnesota School of Public Health, Division of Environmental Health Sciences

^2^University of California, Irvine Program in Public Health

^3^University of Minnesota School of Social Work

Corresponding author:

Austin T Rau

University of Minnesota School of Public Health

rauxx087@umn.edu

**Table S1:** Disciplinary Incident Reporting System (DIRS) incident types used to create the violent incidents category

| Incident type |
| --- |
| Assault |
| Bomb |
| Bomb threat |
| Bullying |
| Cyber bullying |
| Extortion |
| Fighting |
| Gang activity |
| Harassment |
| Hazing |
| Robbery (using force) |
| Terroristic threats |
| Theft |
| Threat/Intimidation |
| Vandalism/Property Related |
| Verbal Abuse |
| Weapon |

**Table S2:** Summary statistics of air pollution concentrations for the schools in the study cohort by levels of urbanicity

| Urbanicity | Total (Median, 25th, 75th percentiles) | Urban (Median, 25th, 75th percentiles) | Rural (Median, 25th, 75th percentiles) |
| --- | --- | --- | --- |
| Pollutant | – | – | – |
| CO (ppm) | 0.28 (0.24, 0.31) | 0.30 (0.28, 0.32) | 0.23 (0.20, 0.26) |
| NO_2_ (ppb) | 5.64 (4.14, 8.70) | 7.68 (5.62, 10.21) | 3.96 (3.16, 4.82) |
| PM_2.5_ (μg/m^3^) | 8.46 (7.73, 9.26) | 8.84 (8.15, 9.60) | 7.84 (7.04, 8.51) |

The 25th percentile, median and 75th percentiles were used to create quartiles of air pollution exposure for total, urban and rural schools respectively.


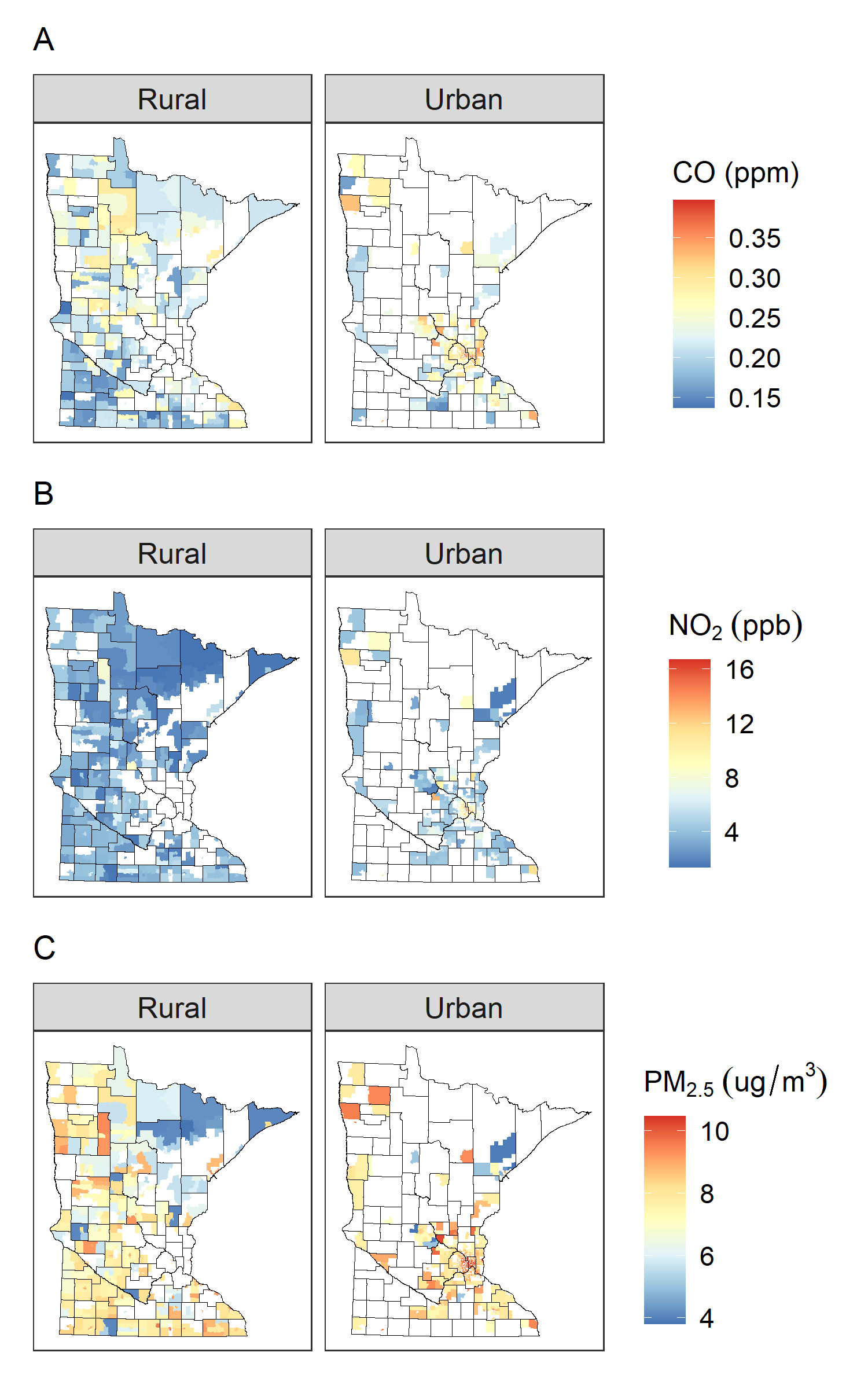


**Figure S1:** 2010 census tract level average air pollution concentrations CO (A), NO_2_ (B), PM_2.5_ (C) stratified by urban and rural census tracts. Unshaded census tracts did not contain a school in our study cohort.

**Table S3:** Crude single pollutant incidence rate ratios (IRR) and incidence rate differences (IRD) estimates describing the association between air pollution and violent and weapon-related incidents for the total student cohort

| Disciplinary incident type | Pollutant (Quartile)^A^ | IRR (95% CI) | IRD (95% CI) |
| --- | --- | --- | --- |
| Violent | PM_2.5_ (Q2) | 0.64 (0.54, 0.76) | -383.03 (-538.77, -227.29) |
|  | PM_2.5_ (Q3) | 0.89 (0.75, 1.06) | -114.58 (-289.03, 59.87) |
|  | PM_2.5_ (Q4) | 1.71 (1.44, 2.03) | 758.86 (504.70, 1013.03) |
|  | NO_2_ (Q2) | 0.75 (0.63, 0.89) | -246.64 (-398.90, -94.38) |
|  | NO_2_ (Q3) | 0.95 (0.80, 1.12) | -53.89 (-219.52, 111.74) |
|  | NO_2_ (Q4) | 1.95 (1.64, 2.32) | 941.51 (677.32, 1205.70) |
|  | CO (Q2) | 1.30 (1.09, 1.55) | 237.82 (75.58, 400.06) |
|  | CO (Q3) | 1.08 (0.91, 1.29) | 62.31 (-81.94, 206.57) |
|  | CO (Q4) | 2.37 (2.00, 2.82) | 1084.5 (837.59, 1331.42) |
| Weapon | PM_2.5_ (Q2) | 0.74 (0.62, 0.88) | -20.59 (-32.57, -8.61) |
|  | PM_2.5_ (Q3) | 0.99 (0.84, 1.16) | -1.01 (-14.05, 12.03) |
|  | PM_2.5_ (Q4) | 1.59 (1.36, 1.86) | 47.04 (30.62, 63.46) |
|  | NO_2_ (Q2) | 0.83 (0.70, 0.99) | -12.28 (-24.21, -0.36) |
|  | NO_2_ (Q3) | 1.10 (0.93, 1.29) | 7.07 (-5.74, 19.88) |
|  | NO_2_ (Q4) | 1.73 (1.46, 2.04) | 53.97 (37.20, 70.74) |
|  | CO (Q2) | 1.23 (1.03, 1.46) | 14.76 (2.27, 27.25) |
|  | CO (Q3) | 1.13 (0.95, 1.34) | 8.50 (-3.28, 20.28) |
|  | CO (Q4) | 1.92 (1.63, 2.27) | 59.61 (43.88, 75.35) |

^A^Quartile 1 for each pollutant is the referent category.

**Table S4:** Adjusted single pollutant incidence rate ratios (IRR) and incidence rate differences (IRD) describing the association between air pollution concentrations with violent and weapon-related incidents. Quartiles of pollution exposure were drawn for the total, urban and rural student cohort cohorts respectively.

| Urbanicity level | Disciplinary incident type | Pollutant (Quartile)^A^ | IRR (95% CI) | IRD (95% CI) |
| --- | --- | --- | --- | --- |
| Total | Violent | PM_2.5_ (Q2) | 0.72 (0.60, 0.85) | -308.74 (-471.87, -145.60) |
|  |  | PM_2.5_ (Q3) | 0.86 (0.73, 1.02) | -150.98 (-323.29, 21.34) |
|  |  | PM_2.5_ (Q4) | 1.47 (1.24, 1.74) | 510.49 (274.92, 746.05) |
|  |  | NO_2_ (Q2) | 0.77 (0.64, 0.91) | -236.69 (-390.70, -82.68) |
|  |  | NO_2_ (Q3) | 1.02 (0.86, 1.21) | 20.97 (-155.78, 197.72) |
|  |  | NO_2_ (Q4) | 1.62 (1.36, 1.94) | 629.16 (384.87, 873.46) |
|  |  | CO (Q2) | 1.26 (1.06, 1.50) | 215.57 (49.77, 381.37) |
|  |  | CO (Q3) | 1.11 (0.93, 1.33) | 94.72 (-61.72, 251.17) |
|  |  | CO (Q4) | 1.93 (1.61, 2.31) | 775.62 (543.2, 1008.05) |
|  | Weapon | PM_2.5_ (Q2) | 0.82 (0.69, 0.98) | -14.26 (-26.73, -1.78) |
|  |  | PM_2.5_ (Q3) | 0.99 (0.84, 1.16) | -0.74 (-13.64, 12.16) |
|  |  | PM_2.5_ (Q4) | 1.42 (1.21, 1.67) | 33.96 (18.44, 49.48) |
|  |  | NO_2_ (Q2) | 0.87 (0.73, 1.03) | -9.94 (-21.84, 1.95) |
|  |  | NO_2_ (Q3) | 1.21 (1.02, 1.42) | 15.23 (1.72, 28.73) |
|  |  | NO_2_ (Q4) | 1.52 (1.28, 1.80) | 38.48 (22.65, 54.31) |
|  |  | CO (Q2) | 1.24 (1.05, 1.48) | 16.07 (3.38, 28.76) |
|  |  | CO (Q3) | 1.20 (1.01, 1.42) | 12.96 (0.44, 25.49) |
|  |  | CO (Q4) | 1.68 (1.41, 1.99) | 44.84 (29.52, 60.15) |
| Urban | Violent | PM_2.5_ (Q2) | 0.87 (0.69, 1.09) | -125.57 (-328.75, 77.61) |
|  |  | PM_2.5_ (Q3) | 1.18 (0.93, 1.48) | 169.49 (-73.01, 411.99) |
|  |  | PM_2.5_ (Q4) | 1.91 (1.50, 2.43) | 874.91 (524.56, 1225.26) |
|  |  | NO_2_ (Q2) | 1.12 (0.89, 1.40) | 90.08 (-94.75, 274.91) |
|  |  | NO_2_ (Q3) | 1.59 (1.26, 2.01) | 457.10 (224.90, 689.30) |
|  |  | NO_2_ (Q4) | 2.67 (2.06, 3.47) | 1288.88 (887.80, 1689.97) |
|  |  | CO (Q2) | 1.10 (0.87, 1.37) | 80.85 (-119.36, 281.05) |
|  |  | CO (Q3) | 1.30 (1.04, 1.63) | 253.78 (32.50, 475.06) |
|  |  | CO (Q4) | 2.32 (1.82, 2.94) | 1113.34 (753.36, 1473.33) |
|  | Weapon | PM_2.5_ (Q2) | 0.94 (0.76, 1.15) | -4.72 (-19.65, 10.21) |
|  |  | PM_2.5_ (Q3) | 1.29 (1.04, 1.59) | 21.19 (3.29, 39.09) |
|  |  | PM_2.5_ (Q4) | 1.61 (1.29, 2.01) | 44.90 (23.45, 66.34) |
|  |  | NO_2_ (Q2) | 1.24 (1.00, 1.53) | 15.25 (0.26, 30.23) |
|  |  | NO_2_ (Q3) | 1.46 (1.18, 1.81) | 29.26 (12.48, 46.04) |
|  |  | NO_2_ (Q4) | 1.91 (1.49, 2.44) | 57.64 (34.34, 80.94) |
|  |  | CO (Q2) | 1.01 (0.82, 1.25) | 1.05 (-14.76, 16.86) |
|  |  | CO (Q3) | 1.23 (1.00, 1.51) | 17.08 (-0.32, 34.47) |
|  |  | CO (Q4) | 1.46 (1.17, 1.83) | 34.70 (13.93, 55.46) |
| Rural | Violent | PM_2.5_ (Q2) | 0.51 (0.39, 0.67) | -698.93 (-1001.96, -395.91) |
|  |  | PM_2.5_ (Q3) | 0.50 (0.39, 0.65) | -711.35 (-1009.16, -413.54) |
|  |  | PM_2.5_ (Q4) | 0.68 (0.51, 0.89) | -464.21 (-796.47, -131.96) |
|  |  | NO_2_ (Q2) | 0.60 (0.46, 0.78) | -559.32 (-859.16, -259.49) |
|  |  | NO_2_ (Q3) | 0.47 (0.36, 0.62) | -734.04 (-1020.67, -447.41) |
|  |  | NO_2_ (Q4) | 0.67 (0.52, 0.87) | -458.79 (-767.23, -150.34) |
|  |  | CO (Q2) | 1.17 (0.89, 1.55) | 129.65 (-99.48, 358.77) |
|  |  | CO (Q3) | 1.61 (1.22, 2.13) | 464.44 (187.21, 741.66) |
|  |  | CO (Q4) | 1.16 (0.87, 1.55) | 122.09 (-115.44, 359.62) |
|  | Weapon | PM_2.5_ (Q2) | 0.76 (0.58, 1.00) | -21.77 (-43.4, -0.15) |
|  |  | PM_2.5_ (Q3) | 0.76 (0.59, 0.99) | -21.59 (-42.46, -0.73) |
|  |  | PM_2.5_ (Q4) | 0.96 (0.74, 1.24) | -3.84 (-26.90, 19.23) |
|  |  | NO_2_ (Q2) | 0.74 (0.56, 0.96) | -24.89 (-46.90, -2.88) |
|  |  | NO_2_ (Q3) | 0.72 (0.55, 0.93) | -26.78 (-48.22, -5.34) |
|  |  | NO_2_ (Q4) | 0.84 (0.66, 1.07) | -15.11 (-36.64, 6.41) |
|  |  | CO (Q2) | 1.19 (0.89, 1.58) | 11.79 (-7.86, 31.43) |
|  |  | CO (Q3) | 1.38 (1.04, 1.83) | 24.25 (3.38, 45.11) |
|  |  | CO (Q4) | 1.31 (0.98, 1.76) | 19.97 (-1.09, 41.04) |

^A^Quartile 1 for each pollutant is the referent category.

**Table S5:** Adjusted multi-pollutant incidence rate ratios (IRR) and incidence rate differences (IRD) describing the association between air pollution concentrations with violent and weapon-related incidents. Quartiles of pollution exposure were drawn for the total, urban and rural student cohort cohorts respectively.

| Urbanicity level | Disciplinary incident type | Pollutant (Quartile)^A^ | IRR (95% CI) | IRD (95% CI) |
| --- | --- | --- | --- | --- |
| Total | Violent | PM_2.5_ (Q2) | 0.69 (0.56, 0.86) | -391.96 (-641.62, -142.29) |
|  |  | PM_2.5_ (Q3) | 0.74 (0.58, 0.95) | -335.01 (-621.87, -48.16) |
|  |  | PM_2.5_ (Q4) | 0.93 (0.69, 1.26) | -83.72 (-453.71, 286.27) |
|  |  | NO_2_ (Q2) | 1.21 (1.01, 1.45) | -64.66 (-267.92, 138.61) |
|  |  | NO_2_ (Q3) | 1.03 (0.84, 1.25) | 153.59 (-113.03, 420.22) |
|  |  | NO_2_ (Q4) | 1.40 (1.10, 1.79) | 424.76 (37.9, 811.62) |
|  |  | CO (Q2) | 0.93 (0.75, 1.16) | 193.53 (7.75, 379.31) |
|  |  | CO (Q3) | 1.16 (0.89, 1.51) | 25.08 (-160.74, 210.90) |
|  |  | CO (Q4) | 1.44 (1.04, 2.00) | 374.36 (96.31, 652.42) |
|  | Weapon | PM_2.5_ (Q2) | 0.76 (0.61, 0.94) | -22.46 (-40.54, -4.38) |
|  |  | PM_2.5_ (Q3) | 0.82 (0.65, 1.03) | -17.21 (-37.62, 3.20) |
|  |  | PM_2.5_ (Q4) | 0.99 (0.75, 1.31) | -0.95 (-26.81, 24.92) |
|  |  | NO_2_ (Q2) | 1.14 (0.95, 1.36) | -0.53 (-15.83, 14.76) |
|  |  | NO_2_ (Q3) | 1.03 (0.85, 1.25) | 22.05 (2.09, 42.01) |
|  |  | NO_2_ (Q4) | 1.22 (0.97, 1.54) | 27.41 (1.03, 53.78) |
|  |  | CO (Q2) | 0.99 (0.80, 1.23) | 10.52 (-3.80, 24.83) |
|  |  | CO (Q3) | 1.31 (1.02, 1.68) | 2.58 (-12.19, 17.35) |
|  |  | CO (Q4) | 1.38 (1.02, 1.88) | 16.78 (-2.48, 36.05) |
| Urban | Violent | PM_2.5_ (Q2) | 0.71 (0.55, 0.91) | -402.77 (-721.69, -83.86) |
|  |  | PM_2.5_ (Q3) | 0.73 (0.54, 0.99) | -370.62 (-744.88, 3.65) |
|  |  | PM_2.5_ (Q4) | 0.90 (0.63, 1.27) | -140.70 (-596.28, 314.89) |
|  |  | NO_2_ (Q2) | 1.30 (0.99, 1.70) | 223.32 (0.87, 445.77) |
|  |  | NO_2_ (Q3) | 1.79 (1.28, 2.50) | 588.93 (257.82, 920.04) |
|  |  | NO_2_ (Q4) | 2.29 (1.52, 3.43) | 959.06 (452.16, 1465.95) |
|  |  | CO (Q2) | 0.95 (0.75, 1.21) | -51.85 (-299.12, 195.42) |
|  |  | CO (Q3) | 1.00 (0.76, 1.31) | -4.00 (-285.28, 277.28) |
|  |  | CO (Q4) | 1.47 (1.08, 2.02) | 491.68 (82.57, 900.78) |
|  | Weapon | PM_2.5_ (Q2) | 0.81 (0.63, 1.03) | -16.88 (-36.84, 3.08) |
|  |  | PM_2.5_ (Q3) | 1.00 (0.75, 1.32) | -0.30 (-25.27, 24.68) |
|  |  | PM_2.5_ (Q4) | 1.14 (0.83, 1.58) | 12.55 (-18.02, 43.12) |
|  |  | NO_2_ (Q2) | 1.34 (1.04, 1.72) | 21.49 (3.61, 39.36) |
|  |  | NO_2_ (Q3) | 1.48 (1.08, 2.04) | 30.76 (6.61, 54.90) |
|  |  | NO_2_ (Q4) | 1.70 (1.16, 2.48) | 44.57 (11.82, 77.32) |
|  |  | CO (Q2) | 0.87 (0.69, 1.09) | -12.24 (-31.89, 7.40) |
|  |  | CO (Q3) | 0.95 (0.74, 1.22) | -4.83 (-27.36, 17.70) |
|  |  | CO (Q4) | 0.98 (0.73, 1.32) | -1.58 (-28.05, 24.88) |
| Rural | Violent | PM_2.5_ (Q2) | 0.65 (0.45, 0.93) | -394.67 (-760.40, -28.93) |
|  |  | PM_2.5_ (Q3) | 0.72 (0.47, 1.10) | -314.08 (-746.00, 117.84) |
|  |  | PM_2.5_ (Q4) | 0.94 (0.56, 1.55) | -73.11 (-627.31, 481.10) |
|  |  | NO_2_ (Q2) | 0.79 (0.54, 1.13) | -258.25 (-671.66, 155.16) |
|  |  | NO_2_ (Q3) | 0.60 (0.39, 0.91) | -485.06 (-925.58, -44.53) |
|  |  | NO_2_ (Q4) | 0.71 (0.43, 1.16) | -351.55 (-865.57, 162.47) |
|  |  | CO (Q2) | 0.93 (0.69, 1.24) | -61.98 (-307.13, 183.16) |
|  |  | CO (Q3) | 1.28 (0.96, 1.71) | 242.97 (-43.22, 529.15) |
|  |  | CO (Q4) | 1.04 (0.77, 1.39) | 32.57 (-226.65, 291.80) |
|  | Weapon | PM_2.5_ (Q2) | 0.95 (0.67, 1.33) | -3.85 (-27.42, 19.73) |
|  |  | PM_2.5_ (Q3) | 1.09 (0.72, 1.65) | 6.47 (-23.57, 36.51) |
|  |  | PM_2.5_ (Q4) | 1.38 (0.85, 2.22) | 26.52 (-13.40, 66.45) |
|  |  | NO_2_ (Q2) | 0.73 (0.51, 1.05) | -27.22 (-60.69, 6.25) |
|  |  | NO_2_ (Q3) | 0.66 (0.44, 0.99) | -35.36 (-71.99, 1.26) |
|  |  | NO_2_ (Q4) | 0.66 (0.41, 1.04) | -35.33 (-75.62, 4.97) |
|  |  | CO (Q2) | 1.04 (0.77, 1.40) | 2.80 (-18.20, 23.79) |
|  |  | CO (Q3) | 1.21 (0.90, 1.62) | 14.50 (-7.67, 36.66) |
|  |  | CO (Q4) | 1.22 (0.90, 1.63) | 14.86 (-7.44, 37.15) |

^A^Quartile 1 for each pollutant is the referent category.


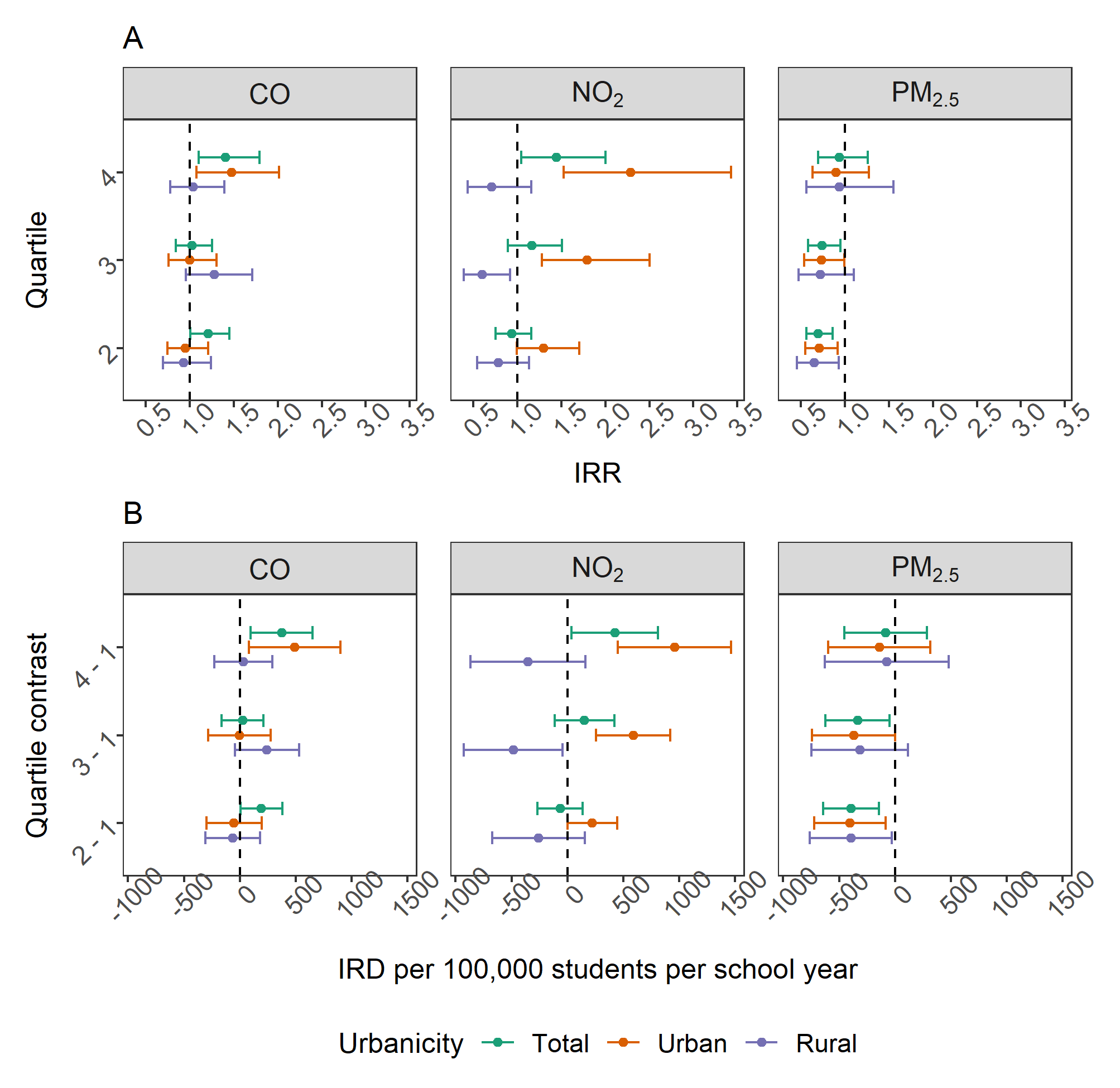


**Figure S2:** Adjusted multi-pollutant incidence rate ratios (IRR) (A) and incidence rate differences (IRD) (B) describing associations between air pollution concentrations with violent incidents with comparisons made between schools in the lowest pollutant quartile (e.g., 1^st^ quartile) and other quartiles of pollution. Pollutant quartiles were drawn within the total, urban, and rural schools respectively.


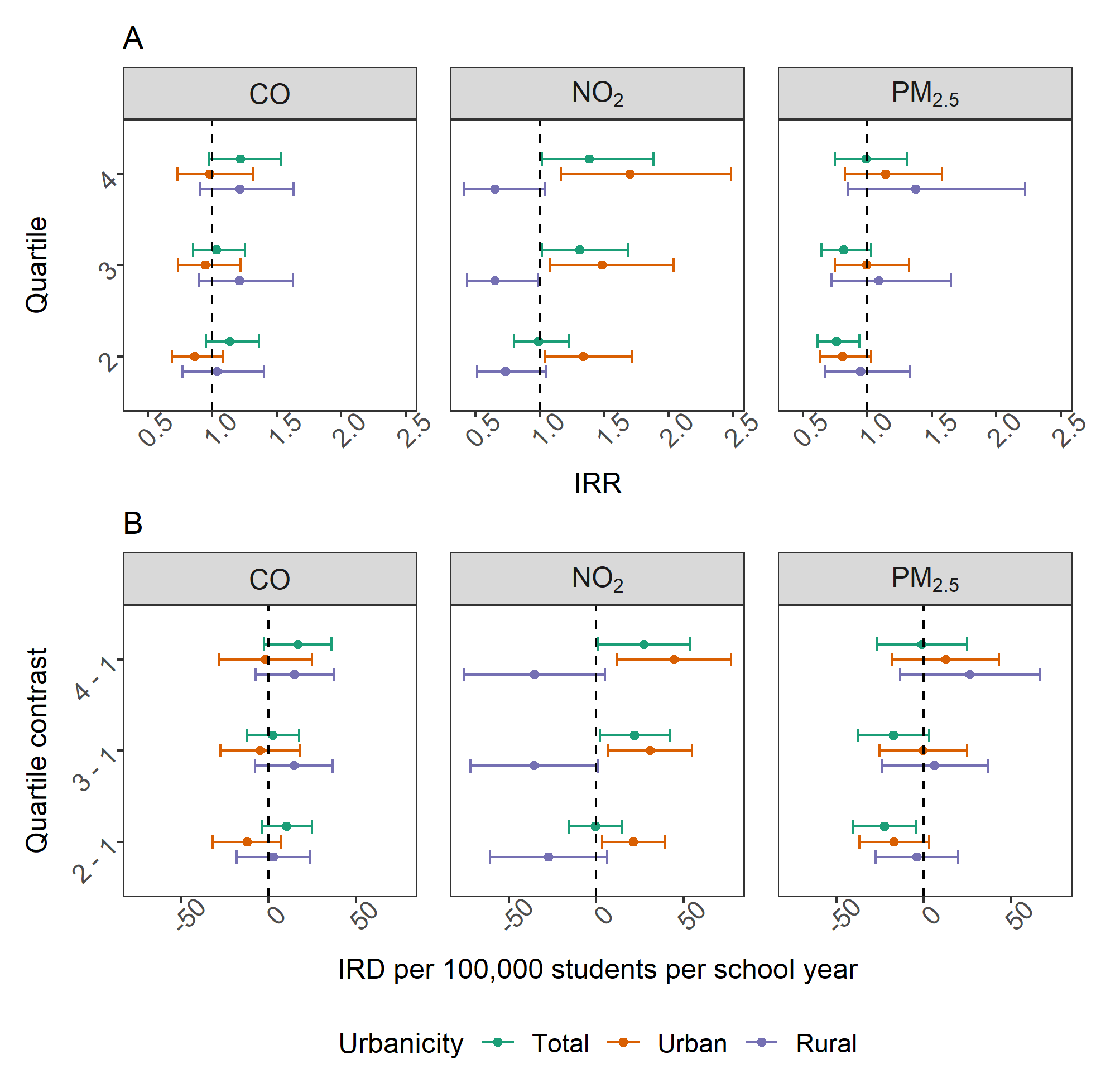


**Figure S3:** Adjusted multi-pollutant incidence rate ratios (IRR) (A) and incidence rate differences (IRD) (B) describing associations between air pollution concentrations with weapon-related incidents with comparisons made between schools in the lowest pollutant quartile (e.g., 1^st^ quartile) and other quartiles of pollution. Pollutant quartiles were drawn within the total, urban, and rural schools respectively.

**Table S6:** Incidence rate ratios (IRR) and incidence rate differences (IRD) describing the association between air pollution exposure and violent incidents in the total study cohort using the socioeconomic (SES) social vulnerability index (SVI) compared to using the overall SVI

| **Pollutant (Quartile)** | **SES SVI model estimates** | | **Overall SVI model estimates** | |
| --- | --- | --- | --- | --- |
|  | **IRR (95% CI)** | **IRD (95% CI)** | **IRR (95% CI)** | **IRD (95% CI)** |
| PM_2.5_ (Q2) | 0.79 (0.66, 0.95) | -207.52 (-366.65, -48.39) | 0.72 (0.60, 0.85) | -308.74 (-471.87, -145.60) |
| PM_2.5_ (Q3) | 0.99 (0.83, 1.17) | -14.63 (-184.53, 155.26) | 0.86 (0.73, 1.02) | -150.98 (-323.29, 21.34) |
| PM_2.5_ (Q4) | 1.61 (1.36, 1.91) | 616.32 (387.96, 844.67) | 1.47 (1.24, 1.74) | 510.49 (274.92, 746.05) |
| NO_2_ (Q2) | 0.86 (0.72, 1.02) | -128.29 (-276.19, 19.6) | 0.77 (0.64, 0.91) | -236.69 (-390.70, -82.68) |
| NO_2_ (Q3) | 1.20 (1.00, 1.44) | 183.33 (0.40, 366.26) | 1.02 (0.86, 1.21) | 20.97 (-155.78, 197.72) |
| NO_2_ (Q4) | 1.81 (1.52, 2.16) | 740.95 (503.56, 978.33) | 1.62 (1.36, 1.94) | 629.16 (384.87, 873.46) |
| CO (Q2) | 1.32 (1.11, 1.58) | 256.68 (92.12, 421.25) | 1.26 (1.06, 1.50) | 215.57 (49.77, 381.37) |
| CO (Q3) | 1.19 (1.00, 1.42) | 153.01 (-4.00, 310.03) | 1.11 (0.93, 1.33) | 94.72 (-61.72, 251.17) |
| CO (Q4) | 2.04 (1.70, 2.45) | 833.14 (602.82, 1063.47) | 1.93 (1.61, 2.31) | 775.62 (543.20, 1008.05) |

**Table S7:** Incidence rate ratios (IRR) and incidence rate differences (IRD) describing the association between air pollution exposure and violent incidents in the total study cohort using an average of air pollution exposure (2008 – 2012) compared to using 2010 air pollution exposure

| **Pollutant (Quartile)** | **Mean Air pollution model** | | **2010 air pollution model estimates** | |
| --- | --- | --- | --- | --- |
|  | **IRR (95% CI)** | **IRD (95% CI)** | **IRR (95% CI)** | **IRD (95% CI)** |
| PM_2.5_ (Q2) | 0.74 (0.62, 0.88) | -283.41 (-446.38, -120.44) | 0.72 (0.60, 0.85) | -308.74 (-471.87, -145.60) |
| PM_2.5_ (Q3) | 0.87 (0.73, 1.04) | -137.42 (-311.82, 36.98) | 0.86 (0.73, 1.02) | -150.98 (-323.29, 21.34) |
| PM_2.5_ (Q4) | 1.50 (1.26, 1.78) | 537.98 (301.70, 774.25) | 1.47 (1.24, 1.74) | 510.49 (274.92, 746.05) |
| NO_2_ (Q2) | 0.81 (0.68, 0.96) | -188.63 (-343.09, -34.16) | 0.77 (0.64, 0.91) | -236.69 (-390.70, -82.68) |
| NO_2_ (Q3) | 1.06 (0.89, 1.26) | 54.19 (-120.60, 228.98) | 1.02 (0.86, 1.21) | 20.97 (-155.78, 197.72) |
| NO_2_ (Q4) | 1.67 (1.40, 2.00) | 655.79 (415.60, 895.98) | 1.62 (1.36, 1.94) | 629.16 (384.87, 873.46) |
| CO (Q2) | 0.94 (0.79, 1.12) | -56.71 (-220.45, 107.02) | 1.26 (1.06, 1.50) | 215.57 (49.77, 381.37) |
| CO (Q3) | 0.97 (0.82, 1.16) | -24.93 (-193.73, 143.87) | 1.11 (0.93, 1.33) | 94.72 (-61.72, 251.17) |
| CO (Q4) | 1.57 (1.31, 1.88) | 554.59 (323.06, 786.12) | 1.93 (1.61, 2.31) | 775.62 (543.20, 1008.05) |


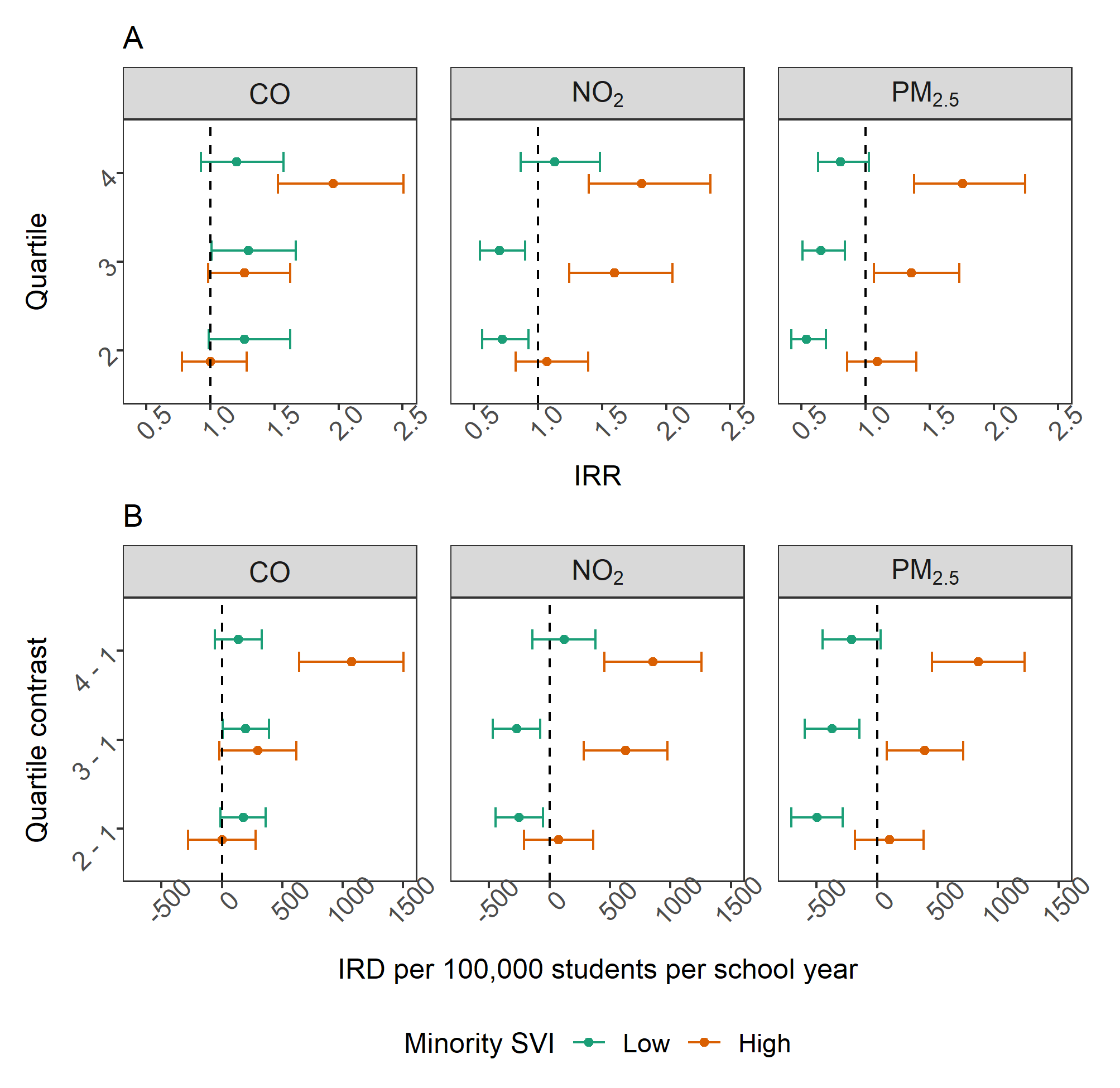
**Figures S4:** Adjusted incidence rate ratios (IRR) (A) and incidence rate differences (IRD) (B) describing associations between air pollution concentrations with violent incidents stratified by the minority status component of the social vulnerability index (SVI). Schools in the high SVI category were located within more racially diverse communities. Comparisons were made between schools in the lowest pollutant quartile (e.g., 1^st^ quartile) and other quartiles of pollution.


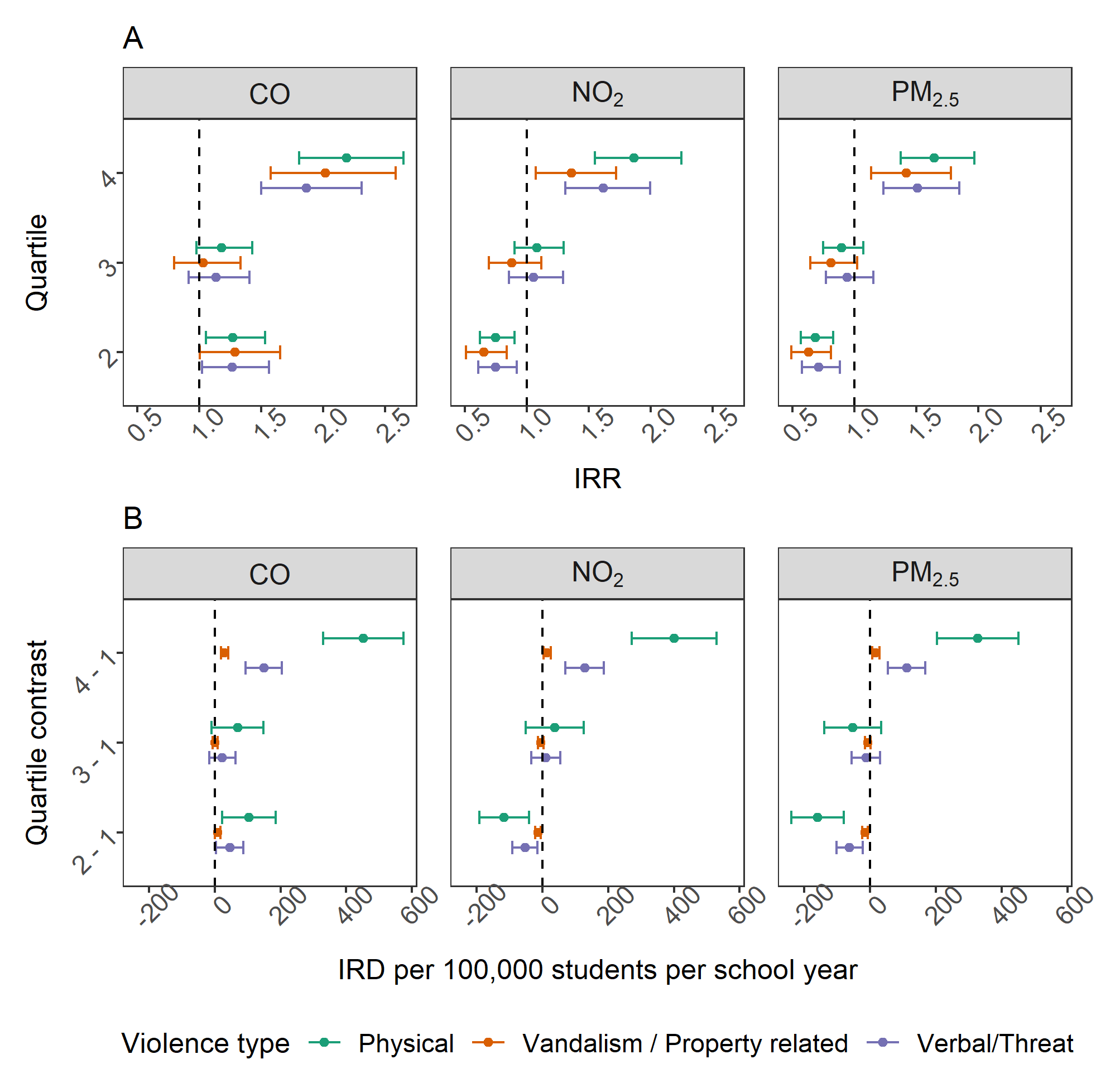


**Figures S5:** Adjusted incidence rate ratios (IRR) (A) and incidence rate differences (IRD) (B) describing associations between air pollution concentrations with physical (fighting or assault or robbery using force), verbal / threat (verbal abuse or threat/intimidation), and vandalism/property related violence. Comparisons were made between schools in the lowest pollutant quartile (e.g., 1^st^ quartile) and other quartiles of pollution.
